# Prevalence, Associated Factors, and Health Expenditures of Noncommunicable Disease Multimorbidity—Findings From Gorakhpur Health and Demographic Surveillance System

**DOI:** 10.3389/fpubh.2022.842561

**Published:** 2022-04-06

**Authors:** Mahendra M. Reddy, Kamran Zaman, Rajaram Yadav, Priyanka Yadav, Kaushik Kumar, Rajni Kant

**Affiliations:** Indian Council of Medical Research-Regional Medical Research Centre (ICMR-RMRC), Gorakhpur, India

**Keywords:** HDSS, health expenditures, India, multimorbidity, noncommunicable diseases

## Abstract

**Background:**

Noncommunicable disease (NCD) multimorbidity throws a unique challenge to healthcare systems globally in terms of not only management of disease, but also familial, social, and economic implications associated with it.

**Objective:**

To assess the prevalence of NCD multimorbidity and its associated risk factors along with health expenditures among adults (≥18 years) living in a rural area.

**Methods:**

A secondary data analysis of the first-round survey done as part of the Gorakhpur Health and Demographic Surveillance Site (GHDSS) was done. Information related to self-reported morbidity and other variables related to sociodemographics and out-of-pocket expenditure (OOPE) was captured using a pretested questionnaire. Multivariable cluster adjusted binomial regression analysis was done to identify factors associated with multimorbidity.

**Results:**

The overall prevalence of NCD multimorbidity was found to be 1.8% (95% CI: 1.7–1.9%). The prevalence of NCD multimorbidity was highest among elderly (≥60 years) [6.0% (95% CI: 5.5–6.5%)] and among women [2.4% (95% CI: 2.3–2.6%)]. Sociodemographic factors, such as age, gender, occupation, education, marital status, religion, caste, and household wealth, were all found to be independently associated with NCD multimorbidity. The median annual OOPE was found to be significantly higher among those with NCD multimorbidity (INR 20,000) compared with those with no NCD (INR 5,000) or having only one NCD (INR 8,000).

**Conclusion:**

Among the adults in GHDSS, about 13 in every 100 were suffering from at least one NCD and around two in 100 were having NCD multimorbidity. Those with NCD multimorbidity spent almost four times higher annual OOPE compared with those without NCDs.

## Introduction

Multimorbidity is simply defined as the “coexistence of two or more chronic conditions in the same individual.” These chronic conditions although are usually noncommunicable diseases (NCDs) in nature but are not limited to NCDs. Some chronic communicable diseases, such as hepatitis B infection, hepatitis C infection, and human immunodeficiency virus infections, also form a part of multimorbidity (1).

Tackling multimorbidity is identified as a health system challenge and identified as a huge problem at present and in coming decades in high-income countries (2). The prevalence of multimorbidity varies over a wide range globally to as high as 90% across the varied age groups (3). In South Asia, the prevalence ranges between 4.5 and 83% (4). The variability may largely be attributed to the way multimorbidity was captured in these studies. Studies reporting the prevalence of multimorbidity in India are limited and mostly done in the elderly age group giving only limited estimates (5–8).

Multimorbidity has been shown to have a greater burden on the healthcare system. Because of multiple conditions, there is an increased risk for hospital admissions, increased medications, all making one invest more in healthcare (9). As seen in individual diseases, premature mortality due to NCDs is on the rise in India which forms the majority of multimorbidity (10). Although age is identified as a single risk factor to multimorbidity, others need to be explored in depth.

The burden of NCDs is on the rise in rural areas and also among younger age groups. The recommendations to screen for NCDs in India in populations <30 years are becoming prominent (11). With the increase in life expectancy in India, people may have to spend more years with multimorbidity and, thus, more healthcare expenses (12). Based on the current demographics in India, identifying multimorbidity is more relevant not only in the elderly, but also in all the adult populations. Studying the epidemiology of multimorbidity, especially in rural India, is essential to tackle this ever-increasing problem at various levels of healthcare.

With this background in this study, we tried to assess the prevalence of NCD multimorbidity and its associated risk factors among adults (≥18 years) living in a rural area. We also tried to see the association of NCD multimorbidity with perceived health status and also OOPE.

## Methodology

We adopted a cross-sectional analytical study design to determine the prevalence of self-reported NCD multimorbidity and its associated factors wherein secondary data analysis of the first-round survey which was done as part of the Gorakhpur Health and Demographic Surveillance Site (GHDSS) project at Gorakhpur, Eastern Uttar Pradesh was used.

The GHDSS conducted its first-round survey from November 2019 to January 2021. GHDSS included a survey in 28 villages belonging to two blocks of the district of Gorakhpur. Apart from the private clinics, the site is catered to by three public healthcare facilities: one primary health center and two community health centers. The majority of the population depends on agriculture for employment. A total of 27,064 households were enlisted during primary household mapping in the GHDSS area. We were able to get consent for data collection from 20,965 households therefore out of 27,064 listed households only 20,965 households were enrolled in the survey. A total of 20,965 households consisting of 120,336 individuals were surveyed during the first round of GHDSS. For this study, we included all the adults (≥18 years) surveyed in the study.

In this GHDSS survey, all the households in the 28 villages were included. If the door was found locked in the first visit, then, it was visited once more on a different day and if found locked again then the house was declared to be “locked.” Informed oral consent was taken from the head of the household and in a case when he/she was not available, it was taken from the available elder member of the household before starting the interview. Preferably, the head of the household and in cases where the head of the household was not available, any other adult member (aged ≥ 18 years) of the household was interviewed using pretested questionnaires to collect data on the health and demography of the household and its members. The data were collected by trained field investigators and was supervised and monitored by trained field supervisors and field scientists. Complete methodology and profile shall be reported as a separate paper.

Information related to self-reported morbidity was captured by asking “Are you suffering from any ailment during the last 15 days?”. In case of more than one morbidity, each morbidity was captured separately and details regarding each morbidity, such as treatment, consultation, and expenditure on ailment, were captured. The ailment name was captured as stated by the participant and then were later coded, which ensured capturing of ailment/disease in a better way. Among the ailments recorded to capture NCD multimorbidity, we took into account the various ailments/diseases which included anemia, cataract, goiter/any thyroid gland disorder, bronchial asthma, chronic obstructive pulmonary disease, gastritis/gastric or peptic ulcer, and digestive system diseases, including hemorrhoids, gallbladder disease/liver disease/pancreatic disease, dementia, mental disorders, stroke, hypertension, ischemic heart disease, diabetes mellitus, kidney diseases, namely, renal failure and renal stones, neoplasms, and musculoskeletal disorders. A person reporting more than one of the included NCD diseases was categorized as having NCD multimorbidity.

The other details collected included age, gender, marital status, occupation, education, family type, religion, and caste. Household wealth was used as a proxy to capture the socioeconomic status and was divided into five quintiles (poorest to wealthiest quintile). The list of assets used in calculating household wealth index included electricity, electric fan, chair, cot/bed, table, sofa, watch/clock, pressure cooker, radio/transistor, sewing machine, animal-drawn cart, mobile/telephone/tablet, television, bicycle, audio-video player, air cooler, computer/laptop, internet connection, refrigerator, mixer/grinder, washing machine, camera/video recorder, motorcycle/ scooter, car, water pump, thresher, tractor, solar panel, mattress, and any other material assets. The steps used in calculating the wealth index were as per the new DHS wealth index calculation (13).

We also determined the coverage of any health insurance and the out-of-pocket health expenditure (OOPE) incurred by the individual. The OOPE was calculated by collecting expenditure status for each ailment, if the expenditure is episodic, it was captured as it is (for example, surgery, outpatient department visits), in case of medications and other monthly recurring expenditures it was captured for monthly and then multiplied by 12 to get annual expenditure; the sum of all expenditures was taken as annual OOPE for that particular ailment. The expenditure was captured as a whole after calculation of OOPE for that particular ailment and then only the total was captured using the ODK tool questionnaire, individual particulars were not captured. The total OOPE per year for an individual was obtained by the total expenditures for all ailments. Similarly, usage of any health facility was also captured for ailments. Thus, we have reported the number who visited any healthcare facility and among them the proportion who incurred OOPE and among those who incurred OOPE the median OOPE [along with interquartile range (IQR)] incurred per year per person in Indian National Rupees (INR). We tried to assess whether there was any difference across the OOPE based on the status of no NCD, one NCD, and NCD multimorbidity (2^+^ NCD).

We also captured their current health status by asking “Currently how do you feel about your health?” with responses captured as either “very good,” “good/fair,” or “poor.” We tried to assess whether the reporting of current health status varied across the NCD and “no NCD” groups.

## Data Entry and Analysis

Data were collected using the Open Data Kit (ODK) software installed in android tablets. Data from the ODK tool were exported into Microsoft Excel format and later coded using STATA version 14.0 for analysis purposes. Continuous variables, such as age, were reported using mean and SD and OOPE was reported using median and IQR. Categorical variables, such as gender, marital status, family type, education status, occupation, religion, caste, and usage of health insurance scheme, were reported as frequency along with percentage. The major outcome of NCD multimorbidity was reported using percentage and its respective 95% CI. NCD multimorbidity along with their 95% CI across different age categories and gender was represented using CI plots and the statistical significance was determined using Pearson's chi-square test. The difference of OOPE across no NCD, one NCD, and 2+ NCD groups were represented using boxplot and the statistical significance was determined using the Kruskal–Wallis test. The difference in reporting of current health status across two groups based on presence or absence of NCD was represented using pie charts and the statistical significance was assessed using the chi-squared test.

The factors associated with NCD multimorbidity were initially assessed using univariate binomial regression analysis. All the associations were reported using prevalence rates (PRs) along with their 95% CI. Multivariable cluster adjusted binomial regression models with all the variables used in univariate analysis were built to determine the adjusted PR with 95% CI. Villages were considered as clusters and adjusting for the cluster was done in the multivariable model. Model significance was reported using the Nagelkerke pseudo-*R*^2^ and corresponding *p*-value for the model which was calculated by using link log under generalized linear model using same variables as used for the multivariable binomial regression model. All the analyses were performed using STATA version 14.0. A *p* < 0.05 was considered to be statistically significant.

## Results

A total of 75,037 individuals aged 18 years and above were included in the final analysis (294 records were excluded as it had missing data for at least one of the variables considered under study). The mean (SD) age of study participants was 37 (15.6) years and 39,071 (52%) were men. Among the participants, 39.1% had no formal schooling and 26% belonged to the wealthiest quintile. The detailed sociodemographic profile is as described in Table 1.

**Table 1:** Details of sociodemographic characteristics of adults residing in HDSS, Gorakhpur (*N* = 75,037).

**Sociodemographic variable**	**Number, *n***	**(%)**
**Age in years**		
18–29	29,048	(38.7)
30–44	23,551	(31.4)
45–59	12,929	(17.2)
≥60	9,509	(12.7)
**Gender**		
Male	39,071	(52.0)
Female	35,920	(47.9)
Transgender	46	(0.1)
**Education**		
No formal schooling	29,382	(39.1)
Primary school or below	9,436	(12.6)
Middle school	10,461	(13.9)
Secondary school	8,386	(11.2)
Higher secondary school/Diploma	9,143	(12.2)
Graduate and above	8,229	(11.0)
**Occupation**		
Unemployed	3,574	(4.8)
Self-employed	9,869	(13.1)
Salaried employee	2,605	(3.5)
Daily wage laborer	21,033	(28.0)
Homemaker	30,318	(40.4)
Student	7,638	(10.2)
**Marital status**		
Never married	16,479	(22.0)
Currently married	53,345	(71.1)
Divorced/Separated/Widowed	5,213	(6.9)
**Family type**		
Nuclear	44,164	(58.9)
Joint/Extended	30,873	(41.1)
**Religion**		
Hindu	71,122	(94.8)
Others	3,915	(5.2)
**Caste**		
Scheduled caste	19,670	(26.2)
Scheduled tribe	1,229	(1.7)
Other backward caste	50,377	(67.1)
Others	3,761	(5.0)
**Household wealth**		
Poorest quintile	11,693	(15.6)
Second poorest quintile	13,254	(17.7)
Mid quintile	14,641	(19.5)
Second wealthiest quintile	15,909	(21.2)
Wealthiest quintile	19,540	(26.0)
**Health insurance/scheme**		
Government funded	7,794	(10.4)
Employer funded	615	(0.8)
Self-funded	2,360	(3.2)
No scheme	64,268	(85.6)
**Perceived health status**		
Very good	17,548	(23.4)
Good / Fair	43,965	(58.6)
Poor	13,524	(18.0)

*HDSS, Health and Demographic Surveillance Site*.

### Noncommunicable Disease Multimorbidity and Its Associated Factors

**Figure 1:**
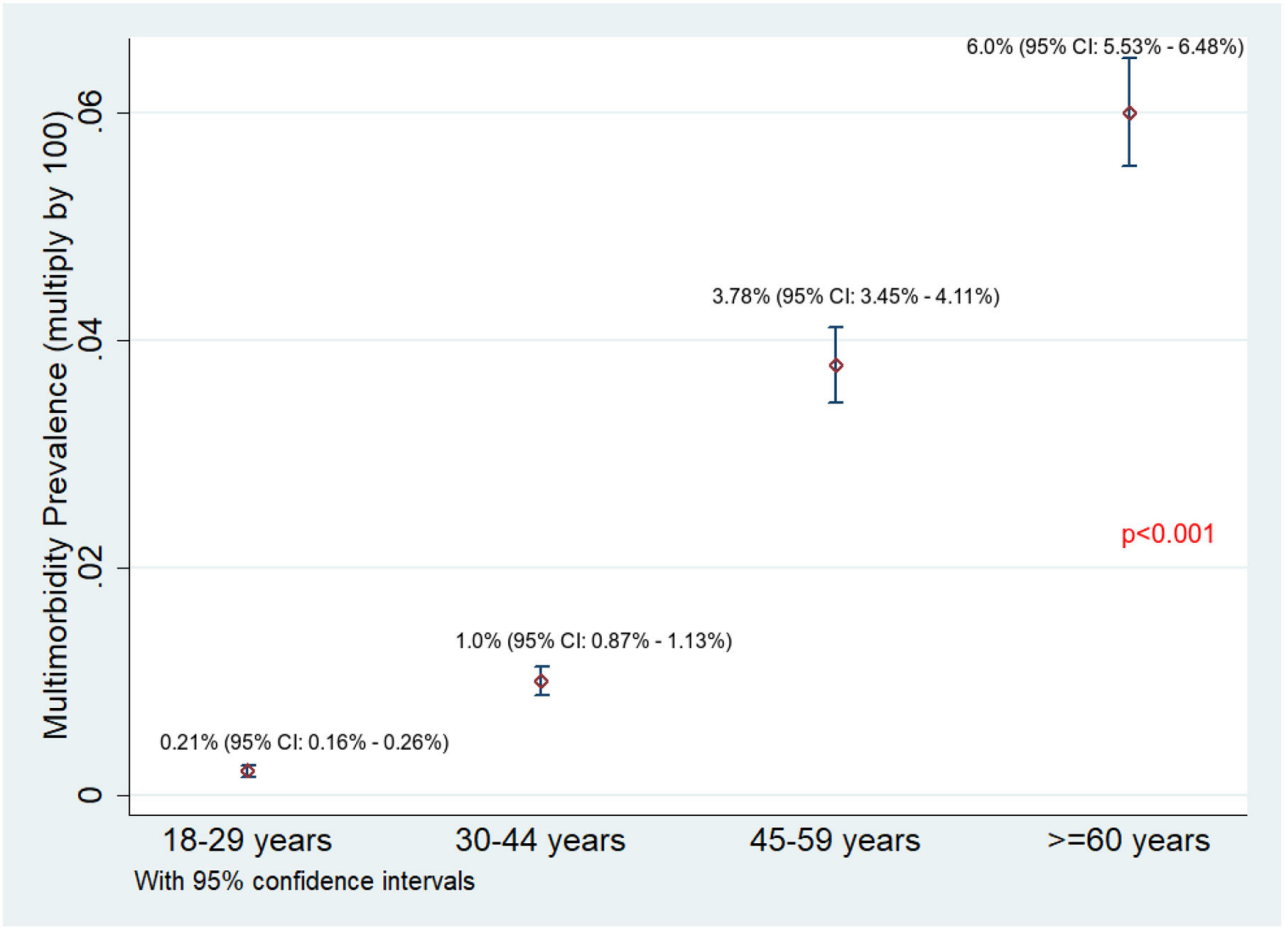
Prevalence of NCD multimorbidity across different age categories among adults residing in HDSS, Gorakhpur (overall, *N* = 75,037; 18–29 years, *n* = 29,048; 30–44 years, *n* = 23,551; 45–59 years, *n* = 12,929; ≥ 60 years, *n* = 9,509).

**Figure 2:**
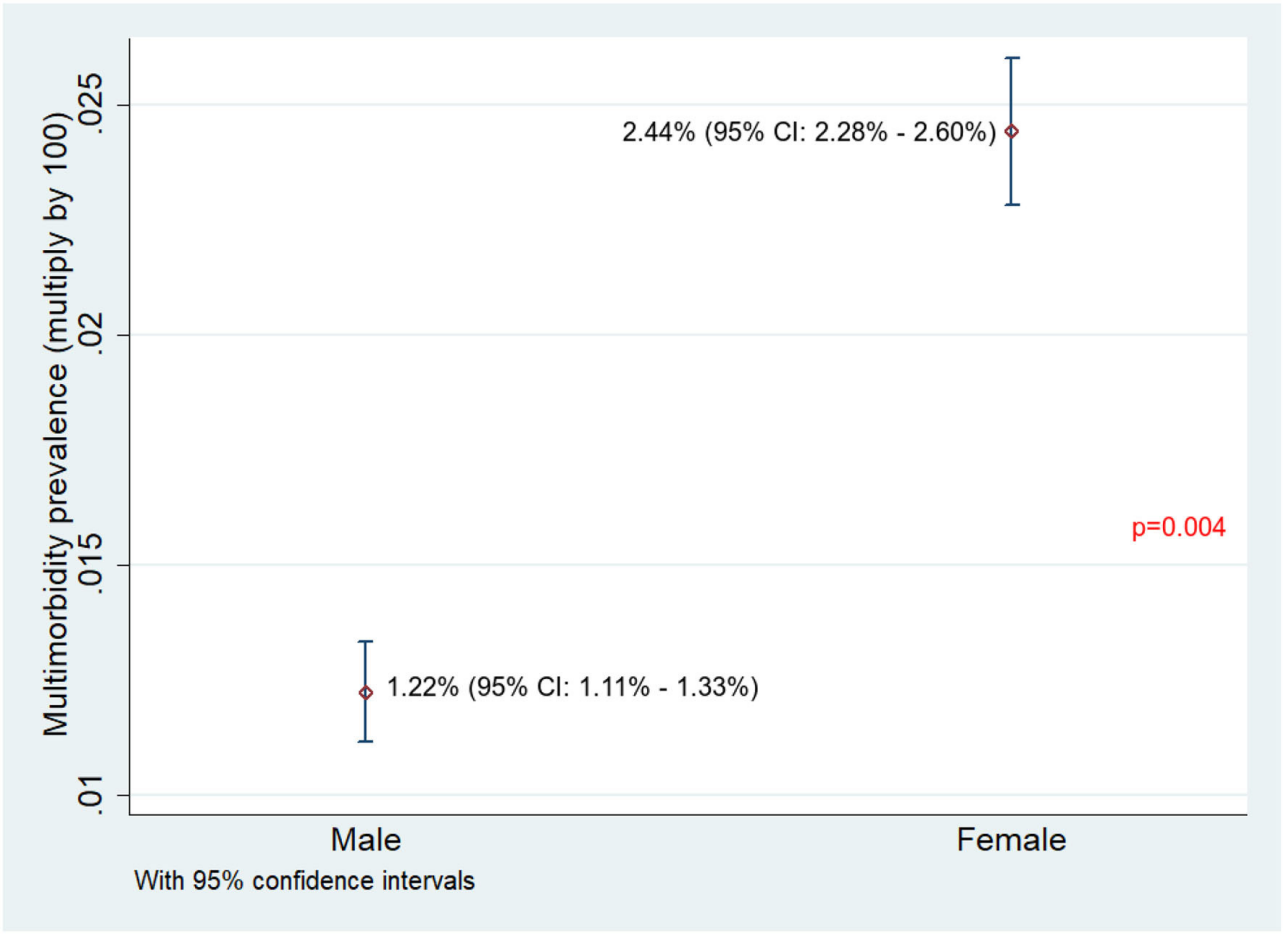
Prevalence of NCD multimorbidity among male and female adults residing in HDSS, Gorakhpur (overall, *N* = 75,037; male, *n* = 39,071; female, *n* = 35,920).

Of 75,037 individuals surveyed, 9,885 (13.2%) reported having at least one NCD. The overall prevalence of NCD multimorbidity (2+ NCD) was found to be 1.81% (95% CI: 1.71–1.90%). The NCD multimorbidity prevalence across different age categories and gender is given in Figures 1, 2, respectively. The prevalence of NCD multimorbidity was highest in the age group of ≥60 years and among women. Sociodemographic factors, such as age, gender, occupation, education, marital status, religion, caste, and household wealth, were all found to be independently associated with NCD multimorbidity. The PRs increased with increasing age and increasing household wealth. The cluster adjusted PRs along with 95% CI of all the independent associations is shown in Table 2.

**Table 2:** Factors associated with NCD multimorbidity among adults residing in HDSS, Gorakhpur (*N* = 75,037).

**Sociodemographic variable**	**Number, *n***	**NCD Multimorbidity, *n* (%)**	**Unadjusted PR (95% CI)**	**Adjusted PR (95% CI)**
Age in years				
18–29	29,048	61 (0.2)	1	1
30–44	23,551	236 (1.0)	**4.8 (3.6–6.3)**	**3.5 (2.5–5.0)**
45–59	12,929	489 (3.8)	**18.0 (13.8–23.5)**	**13.2 (8.8–19.8)**
≥60	9,509	571 (6.0)	**28.6 (22.0–37.2)**	**19.1 (12.9–28.2)**
**Gender**				
Male	39,071	478 (1.2)	1	1
Female	35,920	877 (2.44)	**2.0 (1.8–2.2)**	**1.6 (1.2–2.2)**
Transgender	46	2 (4.3)	3.6 (0.9–13.8)	2.4 (0.8–7.4)
**Education**				
No formal schooling	29,382	840 (2.9)	**4.4 (3.3–5.7)**	1.2 (0.9–1.6)
Primary school or below	9,436	152 (1.6)	**2.5 (1.8–3.3)**	**1.5 (1.1–2.2)**
Middle school	10,461	124 (1.2)	**1.8 (1.3–2.5)**	**1.5 (1.2–1.9)**
Secondary school	8,386	105 (1.3)	**1.9 (1.4–2.7)**	**1.6 (1.2–2.1)**
Higher secondary school/Diploma	9,143	82 (0.9)	1.4 (1.0–1.9)	**1.4 (1.0–2.0)**
Graduate and above	8,229	54 (0.7)	1	1
**Occupation**				
Unemployed	3,574	157 (4.4)	**7.3 (5.8–9.2)**	**3.4 (2.5–5.6)**
Self-employed	9,869	231 (2.3)	**3.9 (3.1–4.8)**	**1.8 (1.4–2.3)**
Salaried employee	2,605	52 (2.0)	**3.3 (2.4–4.6)**	**2.1 (1.5–3.0)**
Daily wage laborer	21,033	127 (0.6)	1	1
Homemaker	30,318	784 (2.6)	**4.3 (3.6–5.2)**	**2.2 (1.7–2.9)**
Student	7,638	6 (0.1)	**0.1 (0.1–0.3)**	0.8 (0.3–2.3)
**Marital status**				
Never married	16,479	22 (0.1)	1	1
Currently married	53,345	1,053 (2.0)	**14.8 (9.7–22.5)**	**2.0 (1.2–3.2)**
Divorced/Separated/Widowed	5,213	282 (5.4)	**40.5 (26.3–62.5)**	**2.0 (1.2–3.5)**
**Family type**				
Nuclear	44,164	727 (1.7)	1	1
Joint/Extended	30,873	630 (2.0)	**1.2 (1.1–1.4)**	0.9 (0.8–1.1)
**Religion**				
Hindu	71,122	1256 (1.8)	1	1
Others	3,915	101 (2.6)	**1.5 (1.2–1.8)**	**1.4 (1.1–1.7)**
**Caste**				
Scheduled caste	19,670	273 (1.4)	1	1
Scheduled tribe	1,229	29 (2.4)	**1.7 (1.2–2.5)**	**1.7 (1.1–2.7)**
Other backward caste	50,377	941 (1.9)	**1.4 (1.2–1.5)**	**1.2 (1.0–1.5)**
Others	3,761	114 (3.0)	**2.2 (1.8–2.7)**	**1.4 (1.0–1.9)**
**Household wealth**				
Poorest quintile	11,693	165 (1.4)	1	1
Second poorest quintile	13,254	184 (1.4)	1.0 (0.8–1.2)	1.1 (0.9–1.3)
Mid quintile	14,641	190 (1.3)	0.9 (0.8–1.1)	1.0 (0.8–1.2)
Second wealthiest quintile	15,909	298 (1.9)	**1.3 (1.1–1.6)**	**1.4 (1.1–1.6)**
Wealthiest quintile	19,540	520 (2.7)	**1.9 (1.6–2.2)**	**1.7 (1.4–2.1)**

*CI, confidence interval; HDSS, Health and Demographic Surveillance Site; NCD, noncommunicable disease; PR, prevalence rate*.

*Model statistics: Pseudo R^2^ = 0.149, p < 0.001*.

*Bold values indicate statistical significance (p < 0.05)*.

### Health Insurance Coverage and OOPE

**Figure 3:**
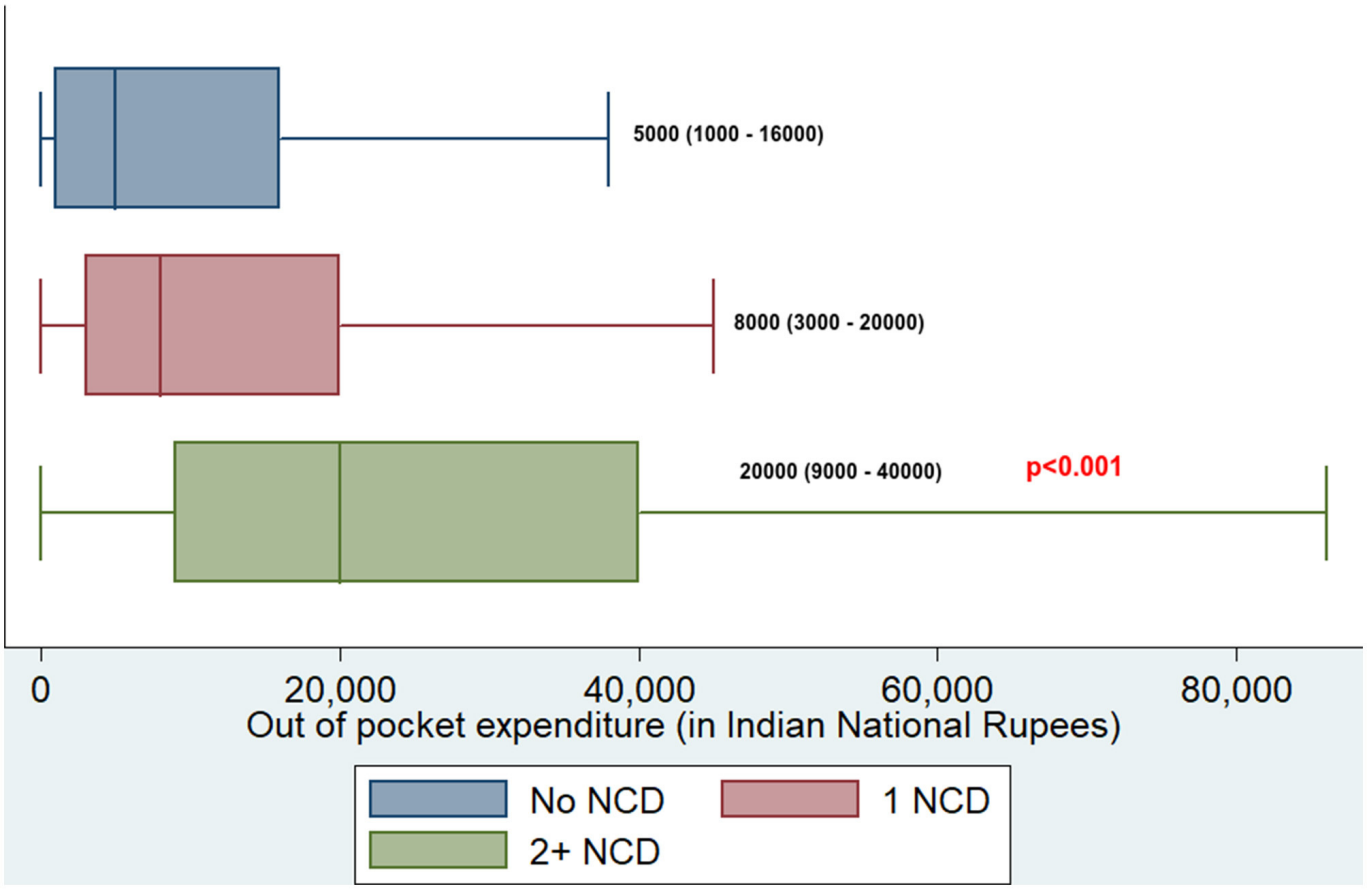
Boxplot depicting median OOPE along with interquartile range among those having no NCD, one NCD, and NCD multimorbidity in adults residing at HDSS, Gorakhpur (overall, *N* = 14,032; no NCD, *n* = 5,078; 1 NCD, *n* = 7,625; 2+ NCD, *n* = 1,329). NCD, noncommunicable disease.

Overall, 10,769 (14.4%) had coverage under any of the health schemes. Among those with NCD multimorbidity, 226 (16.7%) were covered under any of the health schemes. Of 75,037, 14,599 (19.5%) reported using any healthcare facility for their health-related problems. Of the 14,599 who used any healthcare facility, 14,032 (96.1%) reported having incurred OOPE. Among those who incurred OOPE, the median (IQR) overall annual OOPE was found to be INR 8,000 (2,300–20,000). The median annual OOPE was found to be significantly higher among those with NCD multimorbidity compared with those having no NCD or having only one NCD (see Figure 3).

### Noncommunicable Disease and Self-Reported Health Status

**Figure 4:**
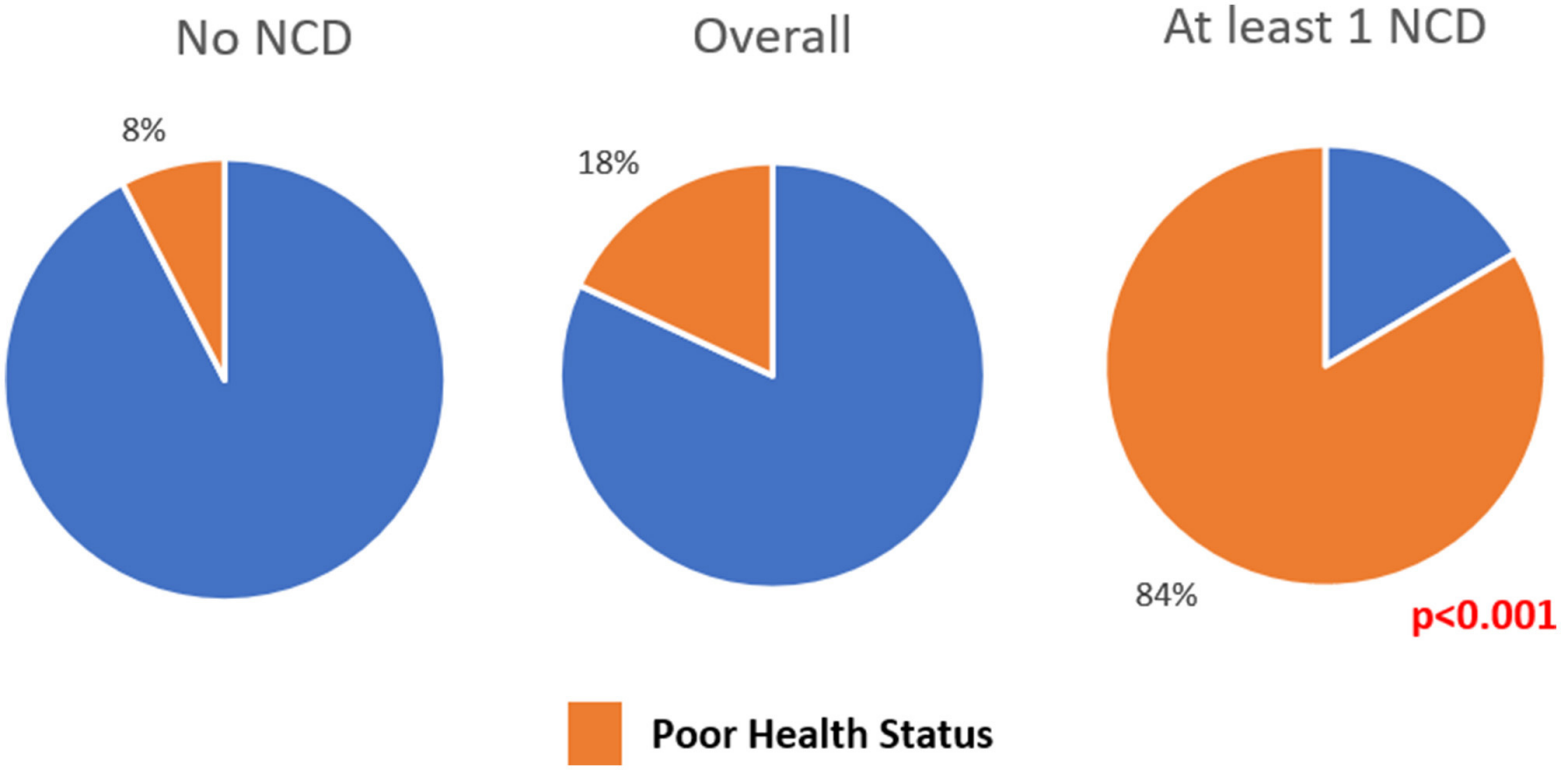
Pie chart depicting the proportion of people reporting current health status as “poor” among adults residing in HDSS, Gorakhpur (overall, *N* = 75,037; no NCD, *n* = 65,152; at least 1 NCD, *n* = 9,885).

Overall, 13,524 (18.0%) felt that their current health status was “poor.” This reporting of “poor” health status was significantly higher among those having at least one NCD compared to those without NCD (see Figure 4). There was not much difference in reporting of “poor” health status among those with one NCD and NCD multimorbidity groups (83.7 vs. 82.7%).

## Discussion

Noncommunicable disease multimorbidity is ever increasing and more so in low- and middle-income countries (LMICs), such as India. Most of the studies on multimorbidity are done in the elderly population and very limited community-based studies are reported from India (5–8). This study covered one of the largest rural populations covering 28 villages of eastern Uttar Pradesh state of India. This study reported that 13% of the adult population have at least one NCD and 1.8% of them have NCD multimorbidity. NCD multimorbidity was found to be 6% among the elderly population (≥60 years).

Studies in India, which included adults more than 18 years, showed the overall prevalence of multimorbidity to be between 9.8 and 34.3% (14–17). Two studies from HDSS in different countries estimated multimorbidity to be 22.8 and 28.7% among 40 years and above population (18, 19). One study done in India in 26 villages in the age group of 20–69 years showed NCD multimorbidity to be 0.7% (20). As there is no standard definition in defining multimorbidity and as also many studies do include other than NCDs in defining multimorbidity the comparisons across studies are difficult. The varied results could also be attributed to reasons, such as studies being conducted across different age groups, the nature of capturing multimorbidity (self-report, measurement, or both), the number of diseases included in capturing multimorbidity, whether it was a community-based survey or facility-based survey and also some regional variations.

With regard to factors associated with NCD multimorbidity, similar to the other studies, we also found NCD multimorbidity to be increasing with age and higher among women and those who were unemployed and those belonging to higher economic status (17–19, 21). Apart from these factors, we found that compared to those who had a graduate level of education those who were literate but below the level of graduate had a higher prevalence of multimorbidity. Similarly, not only unemployed but people employed as either self or salaried class and also homemakers had a higher prevalence of multimorbidity compared to daily wage laborers. With respect to marital status, those who were married had a two times higher prevalence of multimorbid status compared to those who were “never married.” We also found that compared to SC, people belonging to other castes had a higher prevalence of NCD multimorbidity. One of the reasons for these associations can be related to the health-seeking behavior status and thus increased chance of a diagnosis of NCDs. People who are graduates may be busy with their work-life and may have not got themselves screened for NCDs, similarly, those who are economically better may have more access to healthcare facilities and thus increased chances of diagnosing themselves with NCDs. Married people may get themselves screened more due to their social or familial pressure to seek healthcare when required and thus increasing the chance of detection of NCDs.

The higher prevalence among women would call for further integration of national programs directed toward maternal and women's health with NPCDCS. In India, both the programs for NCD and the elderly work in unison and may further be strengthened. There is a need to increase the screening among disadvantaged sections (SCs) and also among the daily wage laborers. As with all the national programs, the need of the hour is to increase the awareness with regard to multimorbidity and the effects that it can have on an individual, family, and at the national level needs to be percolated across all the sections of society.

In this study, we found only 14.4% of adults were having health insurance, of them around three-fourths were public health insurance (72.4%). The health insurance coverage has been on the lower side in India, which contributes to higher OOPE. This study showed that compared to those who do not have NCD, those with NCD multimorbidity incurred four times higher median annual OOPE per person. Although we have not captured the costs as in-patient or outpatient costs; few studies have highlighted an increase in costs in those with NCDs, with the majority being contributed toward medications. We have captured the annual expenditure rather than episodic expenditure which gives an average expenditure toward healthcare in India (22, 23). The increase in expenditure could be due to an increase in seeking healthcare for any of the problems which may be NCD-related or aggravated due to multimorbidity status. Introduction of Pradhan Mantri Jan Arogya Yojana under Ayushman Bharat scheme could address decreasing the OOPE in India (24).

Although multimorbidity itself did not change the perception toward individual health status; having NCD itself, had a significant effect on one's perceived health status with about 84% of those with NCD citing their health status to be “poor” compared to only 8% among those without NCDs. A study centered around the patient satisfaction of the healthcare system toward chronic care in India and Bangladesh showed that 42% were dissatisfied with the health system management toward NCDs (14). Although we have not captured the severity of multimorbidity, this very high number of people with NCDs perceiving their health status to be “poor” needs to be addressed on a high priority. This more so emphasizes the need for counseling among multimorbidity patients to make them aware of their disease status and also its management. There is a hope that this shall be bridged by the Health and Wellness centers which are started across India to strengthen the primary healthcare system as part of the Ayushman Bharath scheme (24).

The increase in the prevalence of NCD multimorbidity in LMICs, such as India, is a cause of public health concern and this needs to be tackled by targeting the root cause of NCD risk factors. A multiprong approach involving cross-cutting interventions (chronic care model) to reduce modifiable risk factors, such as diet, physical activity, and behavioral change in reducing the use of tobacco and alcohol needs to be adopted. There is a need for further research in different settings and high-risk populations along with follow-up surveys to monitor the effectiveness of interventions and also trends.

## Strengths and Limitations

This study has a few strengths. The reporting is from the HDSS which itself accounts for robust data collection and documentation. Instead of listing diseases names and documenting the multimorbidity count, we used a method wherein the individual lists out their ailments in local language which was later coded into diseases, which would have helped in capturing the disease status more accurately. This is one of the largest surveys conducted wherein multimorbidity status was calculated which would have further increased the precision estimates of the outcome measured. We have captured the annual OOPE per person rather than episodic OOPE which would give a new dimension in terms of OOPE incurred in India. We have calculated the household wealth index to determine the economic standards rather than using the income of the family which would have captured the socioeconomic status more accurately. In terms of reporting of association, we have used PRs along with 95% CI which is a more robust measure to report association in the case of cross-sectional studies than using the odds ratio (25). Finally, we have followed the Strengthening the Reporting of Observational studies in Epidemiology (STROBE) guidelines to report this study findings (26).

This study is not without limitations. We have used self-reporting to capture the outcome on multimorbidity which has its inherent bias. Studies in western countries have shown that there is not much difference in capturing multimorbidity *via* self-reporting compared to actual measurements (27, 28). Although we have captured the annual OOPE we have not captured the episodic OOPE and also not accounted for direct and indirect cost estimation. We have captured the current health status using a single question rather than any standard assessment tool which may have introduced bias. As with all cross-sectional studies, temporality between factors associated with multimorbidity cannot be established.

## Conclusion

Among the adults in GHDSS, around 13 out of every 100 are suffering from at least one NCD and around two in 100 are having at least two NCDs. Being women, elderly, married, and belonging to higher economic status were a few factors that were found to be independently associated with NCD multimorbidity. Those with NCD multimorbidity spent almost four times higher annual OOPE compared to those without NCDs. More than four-fifths of those with NCD multimorbidity described their current health status to be “poor.” There is a need for further strengthening of NCD screening, counseling, and integration of various national health programs to tackle NCD multimorbidity.

## Data Availability Statement

The raw data supporting the conclusions of this article will be made available by the authors, without unduereservation.

## Ethics Statement

The study uses secondary data for analysis which do not warrant ethical approval. The primary study involving human participants were reviewed and approved by Institutional Ethical Committee, ICMR-RMRC Gorakhpur.

## Author Contributions

MR and KZ conceived and designed the study. RY, PY, and KK were involved in the acquisition of data. MR, KZ, and RY were involved in analysis and interpretation of data. MR and KZ wrote the first draft. RK revised the manuscript and gave critical inputs. All the authors approved the final version submitted for a journal.

## Funding

Financial support was provided by an extramural grant from the Indian Council of Medical Research (No. VIR/12/2017/ECD-I).

## Conflict of Interest

The authors declare that the research was conducted in the absence of any commercial or financial relationships that could be construed as a potential conflict of interest.

## Publisher's Note

All claims expressed in this article are solely those of the authors and do not necessarily represent those of their affiliated organizations, or those of the publisher, the editors and the reviewers. Any product that may be evaluated in this article, or claim that may be made by its manufacturer, is not guaranteed or endorsed by the publisher.
